# Complement-Mediated Kidney Diseases: Role of Alternative Pathway in Glomerular Inflammation

**DOI:** 10.1016/j.ekir.2025.11.029

**Published:** 2025-12-04

**Authors:** Jonathan Barratt, Peter Garred, Richard A. Lafayette, Hong Zhang, Jürgen Floege

**Affiliations:** 1 Department of Cardiovascular Sciences, University of Leicester, Leicester, UK; 2 Department of Clinical Medicine, Faculty of Health and Medical Sciences, University of Copenhagen, Copenhagen, Denmark; 3 Laboratory of Molecular Medicine, Department of Clinical Immunology, Copenhagen University Hospital Rigshospitalet, Copenhagen, Denmark; 4 Stanford Glomerular Disease Center, Stanford University, Stanford, California, USA; 5 Renal Division, Peking University First Hospital, Beijing, China; 6 Division of Nephrology and Clinical Immunology, RWTH Aachen University, Aachen, Germany

**Keywords:** alternative complement pathway, complement system, complement-mediated kidney disease, emerging therapies, glomerular inflammation

## Abstract

Complement-mediated kidney diseases (CMKDs) comprise a diverse group of rare disorders characterized by the activation of the complement system, leading to glomerular inflammation, kidney injury, and in some cases, kidney failure. Although the contribution of complement activation to disease pathogenesis varies across CMKDs, the alternative complement pathway appears to play a pivotal role in driving inflammation and tissue damage in the kidney by amplifying complement activation, regardless of the initiating complement pathway. A growing body of evidence links the alternative pathway with glomerular inflammation in CMKDs, including key mechanistic insights from preclinical *in vivo* models, the association of alternative pathway components with histologic kidney injury and disease severity, and the efficacy of alternative pathway inhibition in patients with these disorders. With an improved understanding of the mechanisms of alternative pathway overactivation in CMKDs, many novel complement inhibitors targeting the alternative pathway are in clinical development for the management of CMKDs, potentially offering a more precise, better-tolerated, and effective approach than conventional immunosuppressive agents or therapeutics that provide broader inhibition of the common terminal complement pathway. This review summarizes the role of the alternative pathway in the pathogenesis of CMKDs and provides evidence supporting its involvement in glomerular inflammation. In addition, we provide a future perspective on the principles guiding the treatment of glomerular inflammation with therapies that target the alternative pathway.

The complement system is a crucial component of innate immunity and a key modulator of the adaptive immune system, playing a vital role in danger recognition and initiating the inflammatory response.^1^^,^^2^ It facilitates the clearance of pathogens, damaged tissues, and altered host cells through precise and potent opsonic, inflammatory, and cytolytic pathways.^1^^,^^2^ As a powerful driver of immune responses, dysregulation of complement activity has been implicated in the pathogenesis of various disorders, particularly those affecting the kidney.^3^ CMKDs represent a heterogeneous group of rare disorders defined by the activation of the complement system, resulting in progressive glomerular inflammation and injury that often leads to irreversible kidney damage and kidney failure.^4^, ^5^, ^6^ Considering that activation of the complement system occurs in a wide range of kidney disorders with diverse etiologies, CMKDs encompass multiple conditions, including different forms of glomerulonephritides and thrombotic microangiopathies.^5^ Among these disorders, C3 glomerulopathy (C3G) and atypical hemolytic uremic syndrome (aHUS) are considered the prototypical CMKDs, for which there are well-characterized genetic causes of complement activation because of mutations in genes involved in regulating the alternative pathway.^5^^,^^6^ Activation of the complement system also plays a critical role in the pathogenesis of CMKDs in which complement dysregulation is primarily driven by the formation or glomerular deposition of antibodies and/or immune complexes, rather than mutations in genes of the alternative pathway. Such conditions include IgA nephropathy (IgAN), immune complex-mediated membranoproliferative glomerulonephritis (IC-MPGN), membranous nephropathy, lupus nephritis (LN), antineutrophil cytoplasmic autoantibody–associated vasculitis (AAV), anti–glomerular basement membrane disease, and postinfectious glomerulonephritis (Table 1).^5^, ^6^, ^7^, ^8^, ^9^, ^10^, ^11^, ^12^, ^13^, ^14^, ^15^, ^16^, ^17^, ^18^, ^19^, ^20^, ^21^, ^22^, ^23^, ^24^, ^25^, ^26^, ^27^, ^28^, ^29^, ^30^, ^31^, ^32^, ^33^, ^34^, ^35^, ^36^, ^37^, ^38^, ^39^, ^40^, ^41^, ^42^, ^43^, ^44^, ^45^, ^46^, ^47^, ^48^, ^49^, ^50^, ^51^, ^52^, ^53^, ^54^, ^55^, ^56^, ^57^, ^58^, ^59^, ^60^, ^61^, ^62^, ^63^

**Table 1:** Genetic abnormalities and antibodies reported in patients with CMKDs

Complement-mediated kidney disease	Genetic abnormalities		Antibodies	
	Alternative pathway	Other	Alternative pathway	Other
AAV		*A1B8DR3; CTLA4; FcγR; HLA-DPA1; HLA-DPB1; HLA-DQ; HLA-DR4; HLA-DR13(6); HLA-DRB1; HLA-DRB4; PRTN3; PTPN22; SEMA6A; SERPINA1*^11^, ^12^, ^13^, ^14^, ^15^, ^16^, ^17^, ^18^, ^19^		Anti-LAMP2; anti-MPO; anti-PR3^20^, ^21^, ^22^
aHUS	*C3*^a^*; CFB; CFH; CFHR1; CFHR3; CFHR4; CFI; MCP (CD46); THBD*^23^, ^24^, ^25^, ^26^	*DGKε*^26^	Anti-FH^25^^,^^26^	
Antibody-mediated transplant rejection				Anti-blood group A; anti-blood group B; anti-chemokine; anti-chemokine receptor; anti-HLA; anti-MHC class I and II; anti-MICA; anti-MICB; anti-platelet; anti-RAAS^27^^,^^28^
Anti-GBM disease				Anti-type IV collagen α3 chain non-collagenous domain (α3NC1)^29^
C3G	*C3*^a^*; CFB; CFH; CFHR2; CFHR5; CFI; MCP (CD46); THBD (CD141)*^30^, ^31^, ^32^, ^33^, ^34^	*C5; C8; C9*^32^	Anti-C3^a^; anti-C3b^a^; C3NeF; C5NeF; anti-FB; anti-FH^30^^,^^32^, ^33^, ^34^, ^35^	ANA; ANCA/PR3/MPO; anti-C1q; C4NeF; cryoglobulins; anti-dsDNA; anti-GBM; IgA; IgG; IgM; antistreptolysin^32^, ^33^, ^34^^,^^36^
IC-MPGN^b^	*C3*^a^*; CFB; CFH; MCP (CD46); THBD (CD141)*^31^^,^^33^^,^^34^		Anti-C3^a^; anti-C3b^a^; C3NeF; anti-FB; anti-FH^31^^,^^33^, ^34^, ^35^	Anti-C1q; C4NeF; IgA; IgG; IgM^31^^,^^33^^,^^34^^,^^36^
IgAN	*C3*^a^*; CFHR1,3Δ; CFHR5*^37^, ^38^, ^39^, ^40^, ^41^	*CARD9; DEFA; HLA-DQA1; HLA-DQB1; HLA-DRB1; HORMAD2; ITGAM-ITGAX; OSM; LIF; PSMB8; PSMB9; TAP1; TAP2; TNFSF13* (APRIL)*; VAV3*^38^^,^^39^		Anti-βII-spectrin; anti-Gd IgA1; Gd IgA1^42^, ^43^, ^44^
IgAV		*Agt; ACE; C1GALT1; C4; CCL5; CTLA4; eNOS; FVL; HLA-A; HLA-B; HLA-DQA1; HLA-DRB1; HSPA2; ICAM1; IL1β; IL1ra; IL8; MCP1; MEFV; MTHFR; NOS2A; PAX2; PON1; SELP; TGFB1; TNFA; VEGFA*^45^		Anti-Gd-IgA1; Gd IgA1; AECA^46^
LN		*APOL1; FCGR2A; FCGR3A; HAS2; HLA-DR; IRF5; ITGAM (CR3); PDGFRA; STAT4; TNFSF4; TNIP1*^47^	Anti-C3^48,^^a^	Anti-α-actinin; anti-ANXA1; anti-C1q; anti-C4; anti-chromatin; anti-dsDNA; anti-ENO1; anti-histone 2A; anti-ribosomal P; anti-SOD2^48^, ^49^, ^50^, ^51^
MGRS				Monoclonal Ig (including intact Ig, light chains, heavy chains, and cryoglobulins)^52^^,^^53^
MN		*HLA-DQA1; HLA-DRW3; HLA-DRB1; IRF4; NFKB1; PLA2R1*^54^, ^55^, ^56^	Anti-FH^57^^,^^58^	Anti-CNTN1; anti-EXT1; anti-EXT2; anti-FAT1; anti-HTRA1; anti-MPO; anti-NCAM1; anti-NDNF; anti-NELL1; anti-NEP; anti-NTNG1; anti-PCDH7; anti-PCSK6; anti-PLA2R; anti-SEMA3B; anti-THSD7A^59^
PIGN (post-streptococcal glomerulonephritis)	Atypical PIGN: *CFH; CFHR3; CFHR5; CFI*^8^^,^^60^ Acute PIGN: *CFB; CFH*^61^	Atypical PIGN: *C8A; MASP2*^60^	Acute PIGN: Anti-C3b^a^; C3NeF; anti-FB^61^	Anti-C1q; anti-DNase; anti-hyaluronidase; anti-M protein; anti-NAPlr; anti-SpeB; antistreptolysin-O^9^^,^^60^^,^^62^^,^^63^

AAV, ANCA-associated vasculitis; AECA, anti-endothelial cell antibodies; aHUS, atypical hemolytic uremic syndrome; ANA, antinuclear antibody; ANCA, antineutrophil cytoplasmic antibody; ANXA1, annexin A1; APRIL, a proliferation-inducing ligand; C3G, complement 3 glomerulopathy; CMKD, complement-mediated kidney disease; CNTN1, contactin 1; dsDNA, double-stranded DNA; ENO1, enolase 1; EXT, exostosin; FAT1, FAT atypical cadherin 1; FB, factor B; FH, factor H; Gd, galactose-deficient; GBM, glomerular basement membrane; HLA, human leukocyte antigen; HTRA1, high temperature requirement A serine peptidase 1; IC-MPGN, immune complex-mediated membranoproliferative glomerulonephritis; IgAN, IgA nephropathy; IgAV, IgA vasculitis; LAMP2, lysosomal-associated membrane protein 2; LN, lupus nephritis; MGRS, monoclonal gammopathy of renal significance; MHC, major histocompatibility complex; MICA, MHC class I polypeptide-related sequence A; MICB, MHC class I polypeptide-related sequence B; MN, membranous nephropathy; MPO, myeloperoxidase; NAPlr, nephritis-associated plasmin receptor; NCAM1, neural cell adhesion molecule 1; NDNF, neuron-derived neurotrophic factor; NeF, nephritic factor; NELL1, neural epidermal growth factor-like 1 protein; NEP, neutral endopeptidase; NTNG1, netrin G1; PCDH7, protocadherin 7; PCSK6, proprotein convertase subtilisin/kexin type 6; PIGN, postinfectious glomerulonephritis; PLA2R, phospholipase A2 receptor; PR3, proteinase 3; RAAS, renin–angiotensin–aldosterone system; SEMA3B, semaphorin 3B; SOD2, superoxide dismutase 2; SPeB, streptococcal pyrogenic exotoxin B; THSD7A, thrombospondin type 1 domain-containing protein 7A.

a Included because of the central role of C3 in the amplification loop of the alternative pathway.

b Patients with primary (idiopathic) IC-MPGN only.

The contribution of complement activation to disease pathophysiology varies by CMKD, with distinct molecular triggers for complement activation and differential involvement of the classical, lectin, and alternative pathways.^5^ Regardless of the initiating complement pathway, the alternative pathway is a major driver of inflammation and tissue damage, also amplifying the effect of the classical and lectin pathways (Figure 1).^2^^,^^64^, ^65^, ^66^, ^67^, ^68^, ^69^, ^70^, ^71^, ^72^ Therefore, therapeutic inhibition of the alternative pathway may offer improved suppression of the inflammatory and fibrotic processes that contribute to disease progression in CMKDs compared with broader inhibition of the terminal pathway.^73^ Growing insights into the role of alternative pathway overactivation in kidney dysfunction have established the alternative pathway as a viable therapeutic target, underscored by the US Food and Drug Administration approval of the factor B (FB) inhibitor, iptacopan for the reduction of proteinuria in adults with primary IgAN at risk of rapid disease progression and in adults with C3G.^74^, ^75^, ^76^ The approval of iptacopan for IgAN and C3G expands the available repertoire of complement inhibitors for the treatment of CMKDs, which now includes the C5 inhibitors, eculizumab and ravulizumab for aHUS; the C5a receptor antagonist, avacopan for certain forms of AAV; and the C3/C3b inhibitor, pegcetacoplan for C3G and idiopathic (or “primary”) IC-MPGN.^68^^,^^77^, ^78^, ^79^, ^80^

**Figure 1:**
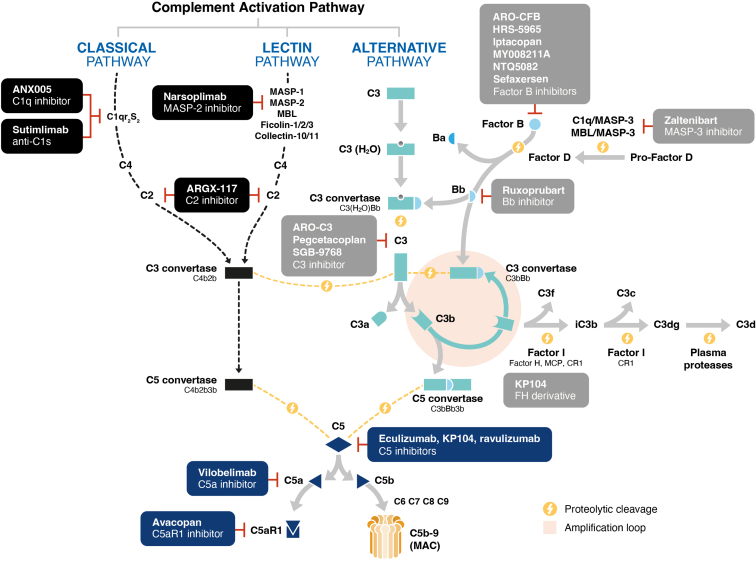
The complement system and complement inhibitors for glomerular inflammation in CMKDs.^2^^,^^64^, ^65^, ^66^, ^67^, ^68^, ^69^, ^70^, ^71^, ^72^ Adapted and reprinted from Tesař *et al.*^65^ under the terms and conditions of the Creative Commons Attribution (CC BY) license (https://creativecommons.org/licenses/by/4.0/). C, complement; CMKD, complement-mediated kidney disease; CR1, complement receptor 1; MAC, membrane attack complex; MASP, MBL-associated serine proteases; MCP, membrane cofactor protein; R, receptor; MBL, mannose-binding lectin.

Herein, we summarize the role of the alternative pathway in the pathogenesis of CMKDs, highlight evidence for its involvement in glomerular inflammation and fibrosis, and offer a perspective on potential principles that could guide future treatment of glomerular inflammation with therapies targeting the alternative pathway.

### Overview of the Complement System and Glomerular Disease

The complement system comprises approximately 50 plasma and cell surface proteins and can be activated via 3 pathways—the classical, lectin, and alternative pathways—that converge at the cleavage of C3 and culminate in the common terminal pathway (Figure 1).^1^^,^^2^^,^^81^ Initiation of the classical and lectin pathways occurs after interaction of pattern-recognition molecules with target structures on pathogenic surfaces or altered endogenous cells.^2^^,^^64^ Specifically, activation of the classical pathway occurs upon binding of the pattern-recognition molecule, C1q with serine proteases, C1r and C1s to various target molecules, including IgG/IgM-containing immune complexes and pathogen-associated molecular patterns.^1^^,^^2^ Similarly, the lectin pathway is activated by the interaction of several pattern-recognition molecules (including mannose-binding lectin, collectin-10, collectin-11, ficolin-1, ficolin-2, and ficolin-3) with associated serine proteases, termed mannose-binding lectin–associated serine proteases (MASPs), when bound to carbohydrate or acetylated groups on altered endogenous cells and pathogens.^2^^,^^64^ In comparison, the alternative pathway is constitutively active at low levels because of spontaneous hydrolysis of C3 (Figure 2a),^82^, ^83^, ^84^ and amplifies both the classical and lectin pathways.^2^ The alternative pathway is the dominant active complement pathway under normal physiological conditions.^2^ However, recent evidence suggests that direct cross-talk with the lectin pathway can activate the alternative pathway in certain conditions, as demonstrated by the cleavage of pro-factor D by mannose-binding lectin–associated serine protease–3^85^, and more recently, by the classical pattern-recognition molecule, C1q.^67^

**Figure 2:**
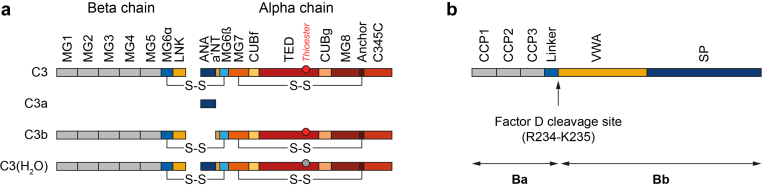
Subunit structure of C3 and factor B.^82^, ^83^, ^84^ (a) Domain composition of C3, C3a, C3b, and C3(H_2_O) molecules. Proteolytic cleavage of C3 by C3 convertases generates the anaphylatoxin C3a and the opsonin C3b. Spontaneous hydrolysis of C3 generates C3(H_2_O), which has functional similarity with C3b. The thioester group of the TED is depicted as a red and gray circle before and after hydrolysis, respectively. Adapted and reprinted from Chen *et al.*^82^ under the terms and conditions of the Creative Commons Attribution (CC BY) license (https://creativecommons.org/licenses/by/4.0/). (b) Domain composition of factor B. Factor D–mediated cleavage of the scissile bond between the linker region and the VWA domain of factor B generates the Ba and Bb molecules. Adapted and reprinted from Hourcade *et al.*^84^ under the terms and conditions of the Creative Commons Attribution (CC BY) license (https://creativecommons.org/licenses/by/4.0/). ANA, anaphylatoxin domain; a’NT, α’-N-terminal segment; C345C, C3, C4, and C5 C-terminal domain; CCP, complement control protein repeat; CUBf, complement C1r/C1s, Uegf, Bmp1 domain of C3f; CUBg, complement C1r/C1s, Uegf, Bmp1 domain of C3g; LNK, linking region; MG, macroglobulin domain; SP, serine protease domain; TED, thioester-containing domain; VWA, von Willebrand factor type A domain.

Despite differing triggering mechanisms, the activation of each complement pathway results in cleavage of C3 and C5 via the assembly of C3 and C5 convertases, respectively.^1^ The C3 and C5 convertases generate the key effector molecules of the complement system, consisting of the opsonin, C3b; anaphylatoxins, C3a and C5a; as well as C5b, an essential component of the membrane attack complex (MAC), also known as C5b-9.^2^ In addition, when the C5b-9 complex is formed, a soluble form of the complex is also generated, which contains the proteins, vitronectin and clusterin.^86^ Although the biological function of the soluble C5b-9 complex remains elusive, it has been shown to be a good marker for evaluating complement activity in plasma and urine.^86^^,^^87^ Notably, the classical/lectin pathway C3 convertase (C4b2b) is formed by the assembly of C4b and C2b, whereas formation of the alternative pathway C3 convertase (C3bBb) requires the association of C3b with factor Bb, generated by factor D–mediated cleavage of factor B (FB) (Figure 2b).^2^^,^^88^ Irrespective of the triggering pathway, the alternative pathway plays a vital role in amplifying complement activation, because C3b generated by all 3 pathways can be incorporated into the alternative pathway C3 convertase, thereby creating the alternative pathway amplification loop (Figure 1).^2^ This amplification loop produces a large number of C3b molecules, which complex with the alternative pathway C3 convertase to form the alternative pathway C5 convertase (C3bBb3b), making this convertase the primary driver of MAC formation.^2^

Complement activation enhances inflammation and potentiates tissue injury via several direct and indirect proinflammatory mechanisms. In addition, it exerts procoagulant and anticoagulant activity through cross-talk with the coagulation system.^1^^,^^2^^,^^89^ As a key effector of the complement system, the assembly of the MAC on cell surfaces directly leads to the formation of pores that disrupt the integrity of the cell membrane and facilitate the lysis of microbes and endogenous tissues.^1^^,^^2^ Further, the C3a and C5a anaphylatoxins further contribute to inflammation by triggering oxidative bursts in phagocytes, and by promoting vasodilation through the induction of histamine release from basophils and mast cells.^2^ Moreover, C5a serves as a potent chemoattractant for T cells.^2^^,^^90^ In the kidney, activated immune cells at sites of inflammation secrete a milieu of profibrotic factors that stimulate the proliferation and differentiation of myofibroblasts from fibroblasts, promoting progressive glomerular and interstitial fibrosis through the production of extracellular matrix components.^91^^,^^92^ Resident glomerular cells, including mesangial, endothelial, and tubular cells, also play an important role in the proinflammatory and profibrotic process by recruiting leukocytes through the production of chemokines and adhesion molecules in response to macrophage-derived factors.^91^ Furthermore, evidence suggests that expression of regulatory complement proteins by tubular cells is limited, making them particularly vulnerable to complement activation in the lumen.^93^^,^^94^ This susceptibility further contributes to tubular injury and subsequent fibrosis in the setting of chronic inflammation.^94^

Regulation of the complement system, particularly of the alternative pathway amplification loop, is therefore crucial for preventing excessive inflammation and tissue damage.^1^^,^^2^ Several regulatory proteins play an important role in controlling the activity of the alternative pathway, predominantly at the level of the alternative pathway C3 convertase.^2^ For example, in the presence of cofactors, including factor H (FH), membrane cofactor protein (i.e., MCP or CD46), and complement receptor 1 (i.e., CR1 or CD35), factor I cleaves and inactivates C3b, thereby reducing or preventing the formation of the alternative pathway C3 convertase.^1^^,^^2^ FH also controls alternative pathway activation in plasma and on tissue surfaces through various other mechanisms, including competing with FB for binding to C3b and inducing the dissociation of the alternative pathway C3 convertase.^2^^,^^5^ On cell surfaces, the alternative pathway is regulated by the decay accelerating factor (i.e., DAF or CD55), which interacts with the C3b and Bb portions of the alternative pathway C3 convertase and promotes dissociation of the complex via its association with C3b.^2^^,^^95^

Conversely, the activity of the alternative pathway is positively regulated by FH-related proteins (FHRs), which bind to extracellular matrix proteins and compete with FH for binding to its ligands, such as C3b; thus promoting formation of the alternative pathway C3 convertase when bound on surfaces.^2^^,^^96^ In addition, recent evidence suggests that FHR1 has other complement-independent, proinflammatory functions, including the induction of inflammasome activation in monocytes at necrotic glomerular sites.^97^

#### Role of Alternative Pathway Overactivation in the Pathogenesis of CMKDs

Among CMKDs, C3G and aHUS are considered the prototypical examples in which overactivation of the alternative pathway plays a primary role in disease pathogenesis because of genetic and/or acquired abnormalities affecting key alternative pathway regulatory proteins (Table 1).^5^^,^^6^^,^^98^^,^^99^ Such genetic abnormalities include gain-of-function mutations in complement activation factors and loss-of-function mutations in key negative regulatory proteins.^5^^,^^98^, ^99^, ^100^ The most commonly reported acquired abnormalities in C3G are the C3 and C5 nephritic factors, which stabilize the alternative pathway convertases, and anti-FH antibodies in aHUS, which disrupt the binding of FH to C3b.^98^, ^99^, ^100^ In C3G, dysregulation of the alternative pathway occurs in both the fluid phase and on glomerular capillary walls, resulting in the glomerular deposition of C3 and its cleavage products. In aHUS, the alternative pathway is typically well-regulated in the fluid phase but overactive on endothelial cell surfaces.^5^^,^^98^ This overactivation likely requires an initial insult to the endothelium, which triggers the disease in patients with a genetic predisposition toward uncontrolled activation of the alternative pathway.^101^

In comparison, overactivation of the alternative pathway in IgAN, IC-MPGN, membranous nephropathy, LN, AAV, anti–glomerular basement membrane disease, and postinfectious glomerulonephritis is predominantly thought to occur secondary to antibodies in systemic circulation and/or deposited in the glomeruli (Table 1).^5^, ^6^, ^7^, ^8^, ^9^^,^^102^ In IgAN, there is evidence of overactivation of the alternative and lectin pathways because of glomerular deposits of proteins associated with these pathways.^103^^,^^104^ Furthermore, recent genome-wide association studies have identified a *CFHR1-3* deletion polymorphism in the *CFH* locus as a factor associated with a reduced risk of developing IgAN.^38^, ^39^, ^40^^,^^104^ Both the alternative and lectin pathways may also play a role in the pathogenesis of nephritis in the closely related disorder, IgA vasculitis.^6^ In primary membranous nephropathy, autoantibodies against the M-type phospholipase A2 receptor (i.e., PLA2R1) have been shown to promote lectin pathway activation^105^; and C3 convertases of the classical/lectin and alternative pathways have been detected in glomerular regions of membranous nephropathy biopsy samples.^106^ All 3 complement pathways are involved in the pathogenesis of anti–glomerular basement membrane disease,^107^ with the involvement of the alternative pathway evidenced by the deposition of FB and properdin along the glomerular capillary wall and by colocalization of FB with C5b-9.^108^ LN is primarily driven by classical pathway activation following the deposition of autoantibodies targeting intrinsic or extrinsic kidney antigens.^5^ Although C4 levels are frequently low in patients with LN,^5^^,^^109^ a disproportionate decline in serum C3 in the absence of lower serum C4 has been significantly correlated with flares of LN, implicating the alternative pathway as a driver of kidney damage.^110^

The role of the alternative pathway in the pathogenesis of IC-MPGN remains poorly understood.^111^ Most cases of IC-MPGN are attributable to secondary causes, including infections, autoimmune diseases, and monoclonal gammopathies.^112^^,^^113^ However, genetic or acquired defects in the regulation of the alternative pathway that are reported in patients with aHUS and C3G have been documented in some patients with primary IC-MPGN.^31^^,^^33^^,^^34^^,^^36^ These findings suggest that alternative pathway overactivation may sometimes be a primary driver of disease. Similarly, some cases of postinfectious glomerulonephritis are associated with defects in the regulation of the alternative pathway, including C3 nephritic factors, anti-FB antibodies, and mutations in *CFH* and *CFHR5*.^8^^,^^61^

In AAV, minimal Ig and complement deposition are seen in the vessels of affected patients.^102^ Rather, AAV is driven by the binding of antineutrophil cytoplasmic autoantibodies to antigens produced by neutrophils in response to priming factors such as infection.^102^ This interaction activates neutrophils, which release factors that activate the alternative pathway, leading to the production of C5a.^102^ In turn, C5a promotes the accumulation and activation of neutrophils, resulting in vascular damage and inflammation.^102^

Beyond the native kidney, overactivation of the alternative pathway contributes to tissue injury and the allograft rejection that is associated with kidney transplantation.^114^^,^^115^ During transplantation, ischemia-reperfusion injury promotes complement activation in the donor kidney, with evidence from animal models suggesting that amplification through the alternative pathway mediates ischemia-related kidney injury.^115^ After transplantation, complement activation contributes to delayed graft function, both chronic and acute graft rejection mediated by T cells and antibodies, and postoperative complications such as immunosuppressive drug toxicity and recurrence of primary disease.^115^ C3G and aHUS are frequently driven by systemic abnormalities in alternative pathway regulatory proteins that often persist in transplant recipients, and are therefore associated with a high risk of primary disease recurrence in the allograft (60%–90% of patients with C3G and 6%–80% of patients with aHUS).^116^, ^117^, ^118^ In C3G, factors influencing the risk of primary disease recurrence include the recipient’s genetic mutation type and the extent of complement activation at the time of transplantation.^118^ However, current evidence is insufficient to reliably assess recurrence risk based on genetic testing, functional complement levels, or the levels of circulating autoantibodies.^118^ In aHUS, the risk of recurrence is influenced by the nature of the affected complement regulator.^6^^,^^117^ Membrane-bound regulatory proteins, such as MCP, are donor-derived; therefore, patients with mutations in genes encoding these factors are less likely to experience primary disease recurrence than patients with mutations in circulating factors.^117^ In addition, the evolution of anti-FH antibodies can stratify the risk of posttransplant recurrence.^6^ Further research is needed to evaluate the contribution of alternative pathway overactivation to posttransplant recurrence across CMKDs.

### Key Features of Glomerular Inflammation and Injury in CMKDs

In patients with CMKDs, glomerular inflammation and injury present along a spectrum, ranging from localized or mild inflammation to diffuse, necrotic, and chronic or irreversible damage (Figure 3).^119^, ^120^, ^121^, ^122^, ^123^, ^124^ Localized or mild inflammation is typically associated with early-stage disease and is characterized by mesangial hypercellularity, exemplified by histopathologic findings in IgAN and class II LN.^120^ As inflammation progresses, glomerular injury may become more diffuse, increasingly compromising the glomerular architecture. Patterns of diffuse injury include diffuse mesangial hypercellularity and endocapillary hypercellularity within glomerular capillary lumina, which may reflect proliferation, inflammatory cell infiltration, and/or endothelial cell swelling.^119^^,^^120^ These patterns of injury are commonly observed in C3G, IgAN, and class III/IV LN^119^^,^^120^^,^^125^ and clinically, may be associated with active disease and proteinuria.^120^^,^^125^ In more severe cases, glomerular inflammation can progress to necrotic injury, characterized by extracapillary hypercellularity, necrosis, and crescent formation, indicating the destruction of the glomeruli.^120^^,^^125^^,^^126^ Crescents are commonly seen in AAV and may also be present in IgAN, C3G, LN, postinfectious glomerulonephritis, and antibody-mediated rejection.^30^^,^^120^^,^^125^, ^126^, ^127^, ^128^, ^129^, ^130^, ^131^, ^132^ The presence of crescents is often accompanied by poor kidney function and an increased risk of progression to kidney failure.^126^^,^^133^ Prolonged inflammation may lead to chronic or irreversible glomerular injury, characterized by fibrosis and glomerular scarring or sclerosis, which permanently compromises the effective function of the glomeruli.^120^^,^^125^ Clinically, this state may be associated with a rapid decline in kidney function, ultimately culminating in kidney failure.^134^

**Figure 3:**
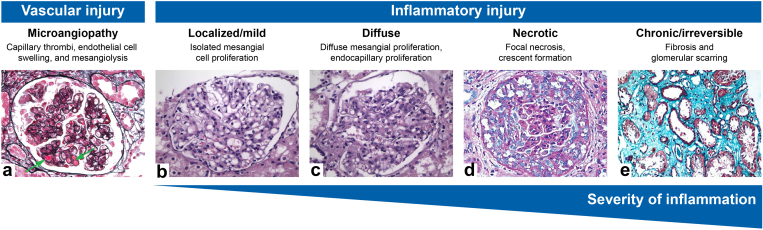
Key patterns of glomerular inflammation and injury in patients with CMKDs. (a) Thrombotic microangiopathy in the glomerular capillary in a patient with LN (green arrows; PASM-Masson). (b) Mild-to-moderate increase in mesangial matrix and mesangial cell proliferation in a patient with IgAN (hematoxylin and eosin). (c) Severe increase in mesangial matrix and mesangial cell proliferation with foci of endocapillary proliferation in a patient with IgAN (hematoxylin and eosin). (d) Full-blown cellular crescent in a patient with crescentic IgAN (periodic acid-Schiff). (e) Severe tubular atrophy and interstitial fibrosis involving >50% of the parenchyma in a patient with IgAN (trichrome). Images reprinted from Zhang *et al.*,^121^ Petrou *et al.*,^122^ Nasri *et al.*,^123^ and Hechanova *et al.*^124^ without modification under the terms and conditions of the Creative Commons Attribution (CC BY) license (https://creativecommons.org/licenses/by/4.0/). CMKD, complement-mediated kidney disease; IgAN, IgA nephropathy; LN, lupus nephritis; PASM, periodic acid-silver methenamine.

As a form of thrombotic microangiopathy, aHUS is predominantly characterized by endothelial injury.^135^ In the glomeruli, such injury manifests as endothelial cell swelling, capillary wall thickening, occlusion of the glomerular capillaries, mesangiolysis, and the deposition of fibrin or fibrinogen in the mesangium and vessel walls.^135^^,^^136^ As such, unlike other forms of glomerulonephritis, patients with aHUS may lack the key mesangial or necrotic changes typically seen in patients with other CMKDs.^135^ However, microangiopathic lesions can also occur in the glomeruli of patients with other CMKDs, including IgAN and LN (Figure 3).^121^^,^^137^^,^^138^

### The Alternative Pathway and Glomerular Inflammation

#### Evidence Linking the Alternative Pathway With Glomerular Inflammation in CMKDs

A growing body of evidence links overactivation of the alternative pathway with glomerular inflammation and fibrosis in CMKDs (Table 2).^97^^,^^139^, ^140^, ^141^, ^142^, ^143^, ^144^, ^145^, ^146^, ^147^, ^148^, ^149^, ^150^, ^151^, ^152^, ^153^, ^154^, ^155^, ^156^, ^157^, ^158^, ^159^, ^160^ In particular, mechanistic data derived from preclinical mouse models have been instrumental in this regard. For example, deletion of either the *FB* or *FD* genes significantly reduces the degree of glomerular inflammation in the MRL/*lpr* mouse model of LN.^149^^,^^150^ Similarly, in a Sendai virus–induced mouse model of IgAN, C3aR deficiency has been shown to reduce mesangial proliferation and expansion, as well as IgA and C3 deposition.^141^ Furthermore, mouse models with abnormalities in the alternative pathway offer insights into the pathogenesis of glomerular injury associated with aHUS and C3G. FH-deficient mice exhibit depleted plasma C3 levels, develop spontaneous membranoproliferative glomerulonephritis with histologic characteristics of C3G, and are sensitized to immune complex–mediated kidney injury.^152^ In addition, FH-deficient mice that express a transgenic mouse FH protein lacking surface recognition domains, functionally equivalent to aHUS-associated human FH mutants,^161^^,^^162^ retain regulation of C3 plasma activation with reduced accumulation of C3 along the glomerular basement membrane and spontaneously develop aHUS but not C3G.^163^ These data indicate that FH mutations specifically impairing surface recognition can result in spontaneous aHUS, whereas FH-deficient mice are predisposed to C3G-like disease.^152^^,^^163^ Interestingly, mice deficient in hepatic FH exhibit low plasma FH and C3 as well as spontaneous mesangial C3 deposition, consistent with C3G, but develop severe C5-dependent thrombotic microangiopathy after a complement-activating trigger.^164^ Therefore, subtotal FH deficiency can result in either spontaneous C3G or aHUS after a complement-activating trigger within the kidney, demonstrating the complexity between FH insufficiency and complement-mediated kidney injury.^164^

**Table 2:** Direct histologic evidence for alternative pathway activity in glomerular inflammation and fibrosis in CMKDs

Complement protein	Indication	Evidence
C3 and C3 activation fragments	C3G	• Low C3/high sC5b-9 levels in plasma correlate with membranoproliferative glomerulonephritis in patients with adult-onset C3G; in the same subgroup of patients, low plasma C3 correlates with endocapillary proliferation, and low C3/normal sC5b-9 correlates with the presence of fibrocellular crescents and interstitial inflammation^139^ • Histopathologic scoring of disease activity in patients with C3G (including the presence of endocapillary and mesangial hypercellularity, capillary neutrophils, cellular crescents, and necrosis) inversely correlates with serum C3 and C5 levels, and positively correlates with plasma sC5b-9^140^
	IgAN	• C3aR deficiency attenuates histologic kidney injury and reduces mesangial proliferation in a Sendai virus-induced mouse model of IgAN^141^ • Plasma iC3b-C3d concentrations correlate with the severity of histologic changes in patients with IgAN, among whom 78% had normal plasma C4d/C4 ratios^142^^,^^143^
Factor B and cleavage factor Bb	AAV	• Factor B is found in the glomerular capillary wall and mesangium of patients with active AAV, and colocalizes with C5b-9 in active glomerular lesions^144^ • Increased levels of plasma Bb are found in patients with active AAV vs patients with remission of AAV, and correlate with the proportion of total and cellular crescents on kidney biopsy^145^ • In patients with AAV, glomerular deposition of Bb correlates with the proportion of crescents and extent of interstitial infiltrates, interstitial fibrosis, and tubular atrophy, while inversely correlating with the proportion of normal glomeruli on kidney biopsy^146^
	C3G	• Inhibition of factor B with iptacopan reduces median C3 deposit score in patients with recurrent C3G after kidney transplant^147^
	IgAN	• In patients with IgAN, higher serum Bb concentration at diagnosis is significantly associated with the presence of E1, S1, and T1/2 lesions, as well as the histologic global optical score and individual vascular and tubular histopathologic indices^148^
	LN	• Homozygous deletion of factor B in the MRL/*lpr* mouse model of LN reduces histologic score for glomerular inflammation and vasculitis compared with factor B-producing mice^149^
Factor D	LN	• Homozygous deletion of factor D in the MRL/*lpr* mouse model of LN reduces glomerular hypercellularity and pathologic score (including polymorphonucleocyte infiltration, hypercellularity, crescent formation, necrosis, presence of thickened basement membrane, and epithelial reactivity) compared with factor D wildtype mice^150^
Factor H	AAV	• Plasma factor H levels inversely correlate with disease activity markers in patients with AAV, including the Birmingham Vasculitis Activity Score, and the proportion of total and cellular crescents on kidney biopsy^151^
	C3G	• Factor H-deficient (*fH*^*–/–*^) mice develop spontaneous membranoproliferative glomerulonephritis with histologic characteristics of C3G and are sensitized to immune complex-mediated kidney injury, which is prevented by simultaneous loss of factor B^152^ • Glomeruli of *fH*^*–/–*^ mice contain deposits of C3b, iC3b, and C3d as well as terminal complement components C5 and C6^153^
	IgAN	• Glomerular factor H staining is significantly reduced in patients with progressive IgAN compared with patients with stable disease^154^
FHR proteins	AAV	• Serum FHR1 concentrations negatively correlate with glomerular filtration rate and are associated with serum markers of inflammation and progressive disease in patients with AAV^97^
	IgAN	• Serum FHR5 is associated with endocapillary hypercellularity and a higher overall histologic MEST score in patients with IgAN^155^ • Plasma FHR1 levels are significantly higher among patients with IgAN compared with healthy persons, and higher among patients with progressive IgAN compared with those with stable disease^155^ • Glomerular FHR5 deposition correlates with glomerular deposits of C3b, iC3b, C3d, and C5b-9, and is associated with progressive disease in patients with IgAN^154^
Properdin	AAV	• Properdin is found in the glomerular capillary wall and mesangium of patients with active AAV, and colocalizes with C3d in active glomerular lesions^144^
	IgAN	• In patients with IgAN, glomerular deposits of properdin are detected in approximately 45% to 95% of cases^157^, ^158^, ^159^, ^160^

AAV, ANCA-associated vasculitis; aHUS, atypical hemolytic uremic syndrome; ANCA, antineutrophil cytoplasmic antibody; C, complement; C3G, complement 3 glomerulopathy; FHR, factor H-related; IgAN, IgA nephropathy; LN, lupus nephritis; MEST, mesangial hypercellularity, endocapillary hypercellularity, segmental sclerosis, and interstitial fibrosis/tubular atrophy; sC5b-9, soluble C5b-9 complex.

Recently, transgenic mice with a C3 gain-of-function amino acid substitution were shown to mirror the clinical phenotype of a single nucleotide substitution in aHUS, which confers resistance of C3b to FH-mediated regulation (D1115N).^165^ Mice with this mutation developed spontaneous chronic thrombotic microangiopathy and low plasma C3 levels with a histologic phenotype consistent with a kidney thrombotic microangiopathy rather than C3G.^165^ This suggests that gain-of-function mutations in C3 can trigger aHUS, likely through extensive and uncontrolled C3b deposition, leading to the subsequent activation of the terminal pathway.^165^ However, a limited number of gain-of-function mutations in C3 that diminish its regulation by FH and/or factor I have been associated with familial C3G.^166^^,^^167^

As previously noted, in patients with CMKDs, the association between glomerular and serologic alternative pathway components with histologic injury and disease severity indicates a role for the alternative pathway in driving kidney damage and influencing disease outcomes (Table 2). For example, in patients with C3G, biomarkers indicative of systemic alternative pathway activation, including low serum C3, low plasma C5, and high plasma levels of soluble C5b-9, have been correlated with histopathologic disease activity scores on kidney biopsy.^140^ Similarly, in a retrospective cohort study of patients with IgAN, serum levels of the positive regulator, FHR5 were associated with endocapillary hypercellularity and higher overall histologic MEST (mesangial hypercellularity, endocapillary hypercellularity, segmental sclerosis, and tubular atrophy/interstitial fibrosis) score as per the Oxford classification of IgAN. In addition, plasma FHR1 levels appeared to be significantly higher among patients with progressive versus stable disease.^155^ Glomerular deposits of FHR5 have been correlated with the deposition of C3b, iC3b, C3d, and C5b-9 in IgAN and are associated with progressive disease.^154^ In contrast, glomerular staining for the negative regulator FH is reduced in patients with progressive IgAN compared with patients with stable disease.^154^

### Managing Glomerular Inflammation in CMKDs

#### Current Strategies for the Treatment of Glomerular Inflammation

Glomerular inflammation in CMKDs is not exclusively driven by complement activation and, historically, it has been managed primarily with systemic glucocorticoids, which aim to suppress the immune response broadly.^112^ Recently, targeted-release budesonide, a locally acting glucocorticoid, was approved by the US Food and Drug Administration to reduce loss of kidney function in adults with primary IgAN at risk of disease progression, and by the European Medicines Agency for the treatment of adults with primary IgAN and a urine protein excretion ≥1.0 g/d (or urine protein-to-creatinine ratio ≥0.8 g/g).^168^^,^^169^ This formulation provides an alternative approach to reducing glomerular inflammation with fewer side effects than systemic glucocorticoids^170^; although further studies are required to evaluate the risk of corticosteroid-associated adverse effects with its long-term use.^171^ An additional strategy for managing glomerular inflammation involves targeting immune cells that play a role in the inflammatory response. Example agents include cytotoxic therapeutics, such as cyclophosphamide; antimetabolites, including mycophenolate mofetil and azathioprine; immunosuppressants, such as calcineurin inhibitors and hydroxychloroquine; and B-cell–depleting therapies, such as rituximab.^112^ However, these immunomodulatory therapies are generally considered to have a slower onset of action compared with the rapid effects that may be achieved by complement inhibition or aggressive steroid dosing.^170^^,^^172^, ^173^, ^174^, ^175^, ^176^, ^177^ In addition, in patients with IgAN, these therapies have shown variable and generally limited efficacy and inconsistent long-term impact on kidney outcomes.^112^^,^^178^, ^179^, ^180^, ^181^, ^182^, ^183^, ^184^, ^185^, ^186^, ^187^

With a growing understanding of the role of complement activation in the pathogenesis of glomerular diseases, anticomplement therapies represent a key targeted strategy for managing glomerular inflammation and injury (Figure 1).^10^^,^^188^ Complement inhibitors are distinct from other immunosuppressive agents because they target pathological processes that are not effectively regulated by other classes of immunosuppressants.^5^ This approach is particularly important given the wide consensus to avoid the use of high-dose and prolonged steroids because of their adverse side effects.^112^

The growing repertoire of complement inhibitors approved in recent years for the treatment of glomerular diseases demonstrates the viability of this approach. For example, the anti-C5 monoclonal antibody, eculizumab has been approved for the treatment of aHUS in the US and the European Union since 2011,^189^^,^^190^ and in China since 2022,^191^ while ravulizumab was approved in the US in 2019, as well as the European Union and Japan in 2020.^192^, ^193^, ^194^ Both drugs target glomerular inflammation at the level of the terminal pathway by preventing the cleavage of C5, thereby preventing the release of C5a and the formation of the MAC.^68^^,^^77^ According to the Kidney Disease: Improving Global Outcomes 2021 guideline, eculizumab can be considered in patients with moderate-to-severe C3G who fail an initial treatment with mycophenolate mofetil plus glucocorticoids.^112^ However, it should be noted that the benefits of C5 blockade in C3G are unestablished.^112^ Small retrospective cohort studies have reported a lack of response to eculizumab therapy in approximately half of treated patients, because C5 inhibition does not address the glomerular C3 deposition that contributes to the disease pathogenesis, which is driven primarily by dysregulation of the alternative pathway.^195^^,^^196^ In 2021 and 2022, the C5a receptor antagonist avacopan was approved by the US Food and Drug Administration and the European Medicines Agency, respectively, for the treatment of adults with severe active AAV (granulomatosis with polyangiitis or microscopic polyangiitis).^78^^,^^79^ Blockade of the C5a receptor by avacopan reduces the proinflammatory effects of C5a, including neutrophil activation and migration.^78^^,^^79^ Underscoring dysregulation of the alternative pathway as a key driver in the pathogenesis of IgAN and C3G, the first-in-class small molecule FB inhibitor, iptacopan, received US Food and Drug Administration approval for proteinuria reduction in adults with primary IgAN at risk of rapid disease progression in 2024, and in adults with C3G in 2025.^74^, ^75^, ^76^^,^^197^ These approvals highlight the potential of inhibiting the alternative pathway as a treatment approach for CMKDs. Most recently, the C3/C3b inhibitor, pegcetacoplan, was approved in the US for the reduction of proteinuria in adult and pediatric patients (≥12 years of age) with C3G or primary IC-MPGN.^80^ By targeting the central C3 molecule and its activation fragment C3b, pegcetacoplan broadly inhibits C3 activation by all 3 initiating complement pathways and the alternative pathway amplification loop, and reduces the generation of effector molecules mediated by the terminal pathway.^80^^,^^198^

#### Targeting the Alternative Pathway in CMKDs – Principles and Future Perspectives

A better understanding of the mechanisms of complement system activation in CMKDs has led to several clinical trials investigating the therapeutic potential of targeting the C3 molecule and the alternative pathway (Table 3).^10^^,^^74^^,^^80^^,^^147^^,^^172^^,^^197^^,^^199^, ^200^, ^201^, ^202^, ^203^, ^204^, ^205^, ^206^, ^207^, ^208^, ^209^, ^210^, ^211^, ^212^, ^213^, ^214^, ^215^, ^216^, ^217^, ^218^, ^219^, ^220^, ^221^, ^222^, ^223^, ^224^, ^225^, ^226^, ^227^, ^228^, ^229^, ^230^, ^231^, ^232^, ^233^, ^234^, ^235^, ^236^, ^237^, ^238^, ^239^, ^240^, ^241^, ^242^, ^243^ Inhibitors of the lectin and terminal pathways are under active clinical investigation for the treatment of CMKDs and are reviewed elsewhere.^5^^,^^10^

**Table 3:** Treatments under clinical investigation for CMKDs that inhibit C3 or the alternative pathway

Agent	Mechanism of action	Indication	Clinical study	Study objectives	Results
C3 inhibitors					
ARO-C3	*C3* RNAi therapeutic^199^	C3G, IgAN	NCT05083364^200^ Phase 1/2a dose-escalating study (ongoing)	Evaluate the safety, tolerability, PK, and PD of ARO-C3 in adult healthy volunteers (Part 1) and adult patients with C3G or IgAN receiving optimized RAASi therapy (Part 2)^200^	Topline results (Part 2)^201^: • At the highest dose tested (3 repeat 400 mg doses), patients with IgAN had a mean reduction of ≥87% in serum C3, ≥89% in Wieslab AP activity assay, and ≥76% in AH_50_ from baseline through week 24 • By week 24, mean reduction in UPCR from baseline was 41% • ARO-C3 was well-tolerated with no serious or severe TEAEs, and no TEAEs leading to study or drug discontinuation
Pegcetacoplan (APL-2)	PEGylated C3/C3b inhibitor^80^	C3G, idiopathic IC-MPGN	NCT05067127 (VALIANT)^202^Phase 3 randomized, double-blind, placebo-controlled trial (completed)	Assess the efficacy and safety of pegcetacoplan on proteinuria reduction vs placebo in adults and adolescents with C3G or idiopathic IC-MPGN^202^	Six-month analysis (randomized controlled period)^203^: • Pegcetacoplan produced a 68% reduction in UPCR vs placebo (P<0.0001), a +6.3 mL/min/1.73m^2^ stabilization of eGFR vs placebo (P=0.03), and a 27-fold higher odds of achieving a ≥2 orders of magnitude reduction in C3c staining intensity vs placebo (P<0.0001) • Pegcetacoplan was well-tolerated with similar rates and severity of TEAEs between pegcetacoplan and placebo groups • There was one death in the pegcetacoplan arm, which was unrelated to the study drug One-year analysis (6-month randomized controlled period and 6-month open-label period)^204^: • Pegcetacoplan maintained a 67% reduction in UPCR and robust stabilization of eGFR • Pegcetacoplan was well-tolerated and there was one mild rejection episode, which was unrelated to the study drug • No patient deaths or allograft losses were reported
			NCT04572854 (NOBLE)^205^ Phase 2 randomized, open-label, controlled trial (ongoing)	Evaluate the safety and efficacy of pegcetacoplan in adult patients with post-transplant recurrence of C3G or idiopathic IC-MPGN^205^	Primary analysis (12 weeks)^206^: • At 12 weeks, 50% and 80% of patients had a reduction of ≥2 orders of magnitude and ≥1 order of magnitude in C3c staining on kidney biopsy from baseline, respectively • Mean C3G histology activity score decreased by >54% in 80% of patients, and 30% had a C3G histologic activity index score of 0 • Patients with ≥1g/g proteinuria at baseline had a median decrease in proteinuria of 54% • Serum C3 increased by 379% from baseline and plasma sC5b-9 decreased by 68% from baseline • Pegcetacoplan was well-tolerated and most TEAEs were mild or moderate in severity; no discontinuations, treatment withdrawals, or deaths were reported One-year analysis^207^: • At 52 weeks, 56% of patients had a reduction of ≥2 orders of magnitude in C3c staining on kidney biopsy from baseline, 44% had no C3c staining on kidney biopsy, 56% had a C3G histologic activity index score of 0, and patients with >1g/g proteinuria at baseline had a median decrease in proteinuria of 56% • Two patients (22%) experienced non-serious rejection episodes and one patient (11%) discontinued treatment for SAE of weight loss • No meningitis cases, graft losses, or deaths were reported
SGB-9768	*C3* RNAi therapeutic^208^	C3G, idiopathic IC-MPGN, IgAN	NCT06786338^209^ Phase 2 open-label study (ongoing)	Evaluate the efficacy and safety of SGB-9678 in adult patients with C3G, idiopathic IC-MPGN, and IgAN^209^	Not yet available
Factor B inhibitors					
ARO-CFB	*CFB* RNAi therapeutic^210^^,^^211^	IgAN	NCT06209177^212^ Phase 1/2a placebo-controlled, dose-escalating study (ongoing)	Evaluate the safety, tolerability, PK, and PD of ARO-CFB in adult healthy volunteers (Part 1) and adult patients with IgAN receiving optimized RAASi therapy (Part 2)^212^	Interim analysis (Part 1)^210^^,^^211^: • Mean reduction of 90% in circulating FB, near complete reduction in Wieslab AP activity assay, and near complete reduction in AH_50_ at multiple dose levels • ARO-CFB was well-tolerated, most TEAEs were mild in severity, no TEAEs led to study or study drug discontinuation, and no infections with encapsulated organisms were reported
HRS-5965	Small molecule factor B inhibitor^213^	IgAN	NCT07014826^214^ Phase 3 randomized, double-blind, placebo-controlled study (ongoing)	Evaluate the efficacy and safety of HRS-5965 in adult patients with primary IgAN receiving optimized RAASi therapy^214^	Not yet available
			NCT06137768^215^ Phase 2 randomized, double-blind, placebo-controlled, dose-escalating study (ongoing)	Evaluate the efficacy and safety of HRS-5965 in adult patients with primary IgAN receiving optimized RAASi therapy^215^	Not yet available
Iptacopan (LNP023)	Small molecule factor B inhibitor^74^^,^^197^	AAV	NCT06388941^216^ Phase 2 randomized, double-blind, placebo-controlled trial (ongoing)	Evaluate the efficacy and safety of iptacopan plus rituximab vs rituximab for the treatment of newly diagnosed or relapsed adult patients with active GPA or MPA^216^	Not yet available
		aHUS	NCT04889430 (APPELHUS)^217^ Phase 3 single-arm, open-label study (ongoing)	Evaluate the efficacy and safety of iptacopan in adult patients with aHUS not previously treated with complement inhibitor therapy^217^	Not yet available
			NCT05935215^218^ Phase 3 single-arm, open-label study (ongoing)	Evaluate the efficacy and safety of switching from anti-C5 antibody to iptacopan treatment in adult patients with aHUS^218^	Not yet available
		C3G^a^	NCT04817618 (APPEAR-C3G)^219^ Phase 3 randomized, double-blind, placebo-controlled trial (ongoing)	Evaluate the efficacy and safety of iptacopan in adults and adolescents with native C3G receiving optimized RAASi therapy^219^	Six-month analysis^220^: • Reduction of 35% in 24-hour UPCR at 6 months vs placebo (P=0.0014) • Numerical improvement in eGFR of +2.2. mL/min/1.73m^2^ from baseline at 6 months vs placebo (P=0.324) • 7-fold increase in odds of meeting composite endpoint (≥50% reduction in proteinuria and stable eGFR) at 6 months vs placebo (OR:7.1; 95% CI: 1.4, 35.7; P=0.0166) • Iptacopan had a favorable safety profile with no deaths, no treatment discontinuations because of TEAEs, and most TEAEs were mild to moderate in severity One-year analysis^221^: • Reduction in 24-hour UPCR with iptacopan was sustained at 12 months • Sustained improvement in patients meeting composite renal endpoint of ≥50% reduction in UPCR plus ≤15% reduction in eGFR at 12 months with iptacopan vs placebo (44%) compared with iptacopan switch (25%) • Iptacopan had a favorable safety profile with no new safety signals
			NCT03832114^147^ Phase 2 single-arm open-label study (completed)	Evaluate the efficacy, safety, and PK of iptacopan in adult patients with native C3G and C3G recurrence after kidney transplant^147^	Final analysis (12 weeks)^147^: • Reduction in UPCR of 45% from baseline in the native cohort (P=0.0003) • In the kidney transplant cohort, median C3 deposit score decreased by 2.5 vs baseline (P=0.03) • Iptacopan was well-tolerated and most AEs were mild or moderate
		Idiopathic IC-MPGN	NCT05755386 (APPARENT)^222^ Phase 3 randomized, double-blind, placebo-controlled trial (ongoing)	Evaluate the efficacy and safety of iptacopan in adults and adolescents with idiopathic IC-MPGN receiving optimized RAASi therapy^222^	Not yet available
		IgAN^b^	NCT04578834 (APPLAUSE-IgAN)^223^ Phase 3 randomized, double-blind, placebo-controlled trial (ongoing)	Demonstrate the superiority of iptacopan vs placebo at reducing proteinuria and slowing disease progression in adult patients with primary IgAN receiving optimized RAASi therapy^223^	Interim analysis (9 months)^172^: • Mean reduction of 38% in 24-hour UPCR at 9 months vs placebo (P<0.001) • Iptacopan was well-tolerated and had a favorable safety profile with most TEAEs of mild or moderate severity • Serious TEAEs were reported in 18 (8%) and 11 (5%) of patients in the iptacopan arm and placebo arm, respectively • No increased risk of infection was observed in the iptacopan group
			NCT06994845^224^ Phase 3 single-arm open-label study (ongoing)	Assess the efficacy, PK, safety, and tolerability of iptacopan in pediatric patients with primary IgAN^224^	Not yet available
			NCT06797518^225^ Phase 2 single-arm open-label biopsy study (ongoing)	Evaluate structural and functional changes in the kidneys of adult patients with IgAN receiving iptacopan and optimized RAASi therapy^225^	Not yet available
			NCT03373461^226^ Phase 2 randomized, double-blind, placebo-controlled, dose-ranging study (completed)	Assess the efficacy and safety of iptacopan in adult patients with primary IgAN^226^	Final analysis^226^: • At 3 months, mean reduction of 23% from baseline in 24-hour UPCR with iptacopan 200 mg twice daily, increasing to a 40% reduction at 6 months • eGFR was stabilized at all dose levels through month 6 of iptacopan treatment • Patients treated with iptacopan had sustained reductions in plasma Bb, serum Wieslab AP activity assay, and urinary sC5b-9 from baseline through month 3 • Iptacopan was well-tolerated and most AEs were mild or moderate in severity • No deaths, treatment-related SAEs, or infections attributed to encapsulated bacteria were reported with iptacopan treatment
		LN	NCT05268289^227^ Phase 2 randomized, double-blind, dose exploration, placebo-controlled trial (ongoing)	Evaluate the efficacy, safety, and tolerability of iptacopan vs placebo in addition to supportive care in adult patients with active Class III/IV ± Class V LN^227^	Not yet available
MY008211A	Small molecule factor B inhibitor^228^	IgAN	NCT06687174^229^ Phase 2 randomized, double-blind, placebo-controlled trial (ongoing)	Evaluate the efficacy and safety of MY008211A in adult patients with IgAN^229^	Not yet available
NTQ5082	Small molecule factor B inhibitor^230^	IgAN	NCT06982040^230^ Phase 2 randomized, double-blind, placebo-controlled trial (ongoing)	Assess the efficacy and safety of NTQ5082 in adult patients with IgAN receiving optimized RAASi therapy	Not yet available
Ruxoprubart (NM8074)	Humanized anti-factor Bb monoclonal antibody^231^	AAV	NCT06226662^232^ Phase 2 randomized, double-blind, placebo-controlled trial (ongoing)	Assess the safety, tolerability, and efficacy of ruxoprubart in combination with cyclophosphamide/ azathioprine or rituximab plus corticosteroids in adult patients with AAV^232^	Not yet available
		aHUS	NCT05684159^233^ Phase 2 non-randomized, sequential assignment, open-label study (ongoing)	Determine if ruxoprubart results in remission from TMA in treatment-naïve adult patients with aHUS^233^	Not yet available
		C3G	NCT05647811^234^ Phase 1b non-randomized, sequential assignment, open-label, dose-escalation study (ongoing)	Evaluate the safety, efficacy, and immunogenicity of ruxoprubart in adult patients with C3G^234^	Not yet available
		IgAN	NCT06454110^235^ Phase 2 single-arm, open-label study (ongoing)	Evaluate the safety and efficacy of ruxoprubart in reducing proteinuria in adult patients with IgAN^235^	Not yet available
Sefaxersen (IONIS-FB-L_Rx_/RO7434656)	Antisense oligonucleotide of *CFB* mRNA^236^	IgAN	NCT05797610 (IMAGINATION)^237^ Phase 3 randomized, double-blind, placebo-controlled trial (ongoing)	Evaluate the efficacy, safety, and PK of sefaxersen in adult patients with primary IgAN at high risk of disease progression despite optimized RAASi therapy^237^	Not yet available
			NCT04014335^238^ Phase 2a single-arm, open-label study (completed)	Evaluate the efficacy and safety of sefaxersen and evaluate the effect of sefaxersen on plasma factor B levels, AH_50_, and CH_50_, in adult patients with primary IgAN^238^	Primary analysis (29 weeks)^239^: • Mean reduction in 24-hour proteinuria of 43% from baseline • eGFR was maintained from baseline • Selective reductions were achieved in plasma FB, plasma Bb, serum AP activity, urinary Ba, and urinary sC5b-9 without changes in serum CH_50_; • There was one treatment emergent serious AE not related to study drug and 3 patients (13%) experienced reversible ALT elevations without a change in serum bilirubin
Factor H derivatives					
KP104	Recombinant bifunctional fusion protein containing two moieties of five SCRs of FH (FH_1–5_) and anti-C5 monoclonal antibody^240^	C3G, IgAN	NCT05517980^241^ Phase 2 randomized, sequential assignment, open-label study (ongoing)	Evaluate the efficacy, safety, PK, and PD of KP104 in adult patients with IgAN or C3G receiving optimized RAASi and/or SGLT2i therapy^241^	Not yet available
MASP-3 inhibitors					
Zaltenibart (OMS906)	Humanized anti-MASP-3 antibody that blocks activation of pro-FD^242^	C3G, idiopathic IC-MPGN	NCT06209736^243^ Phase 2 single-arm, open-label study (ongoing)	Assess the safety, tolerability, PK, PD, and preliminary efficacy of zaltenibart in adult patients with C3G or idiopathic IC-MPGN^243^	Not yet available

Data derived from www.clinicaltrials.gov (accessed June 23, 2025). Included studies were limited to investigational trials only (phase 1–3) with a study start date on or after January 1, 2019. Extension studies were not included.

AH_50_, alternative pathway hemolytic activity 50%; aHUS, atypical hemolytic uremic syndrome; AAV, ANCA-associated vasculitis; AE, adverse event; ALT, alanine aminotransferase; ANCA, antineutrophil cytoplasmic autoantibody; AP, alternative pathway; C3G, complement 3 glomerulopathy; CH_50_, classical pathway hemolytic activity 50%; eGFR, estimated glomerular filtration rate; FDA, US Food and Drug Administration; GPA, granulomatosis with polyangiitis; IC-MPGN, immune complex-mediated membranoproliferative glomerulonephritis; IgAN, IgA nephropathy; LN, lupus nephritis; MASP-3, mannan-associated lectin-binding serine protease-3; MN, membranous nephropathy; MPA, microscopic polyangiitis; PD, pharmacodynamics; PEG, polyethylene glycol; PK, pharmacokinetics; RAASi, renin–angiotensin–aldosterone system inhibitor; SAE, serious adverse event; SCR, short consensus repeat; sC5b-9, soluble C5b-9 complex; SGLT2i, sodium-glucose cotransporter-2 inhibitor; TEAE, treatment-emergent adverse event; TMA, thrombotic microangiopathy; UPCR, urine protein–creatinine ratio.

a Iptacopan received regulatory FDA approval for the reduction of proteinuria in adults with C3G in March 2025.^74^^,^^76^

b Iptacopan received accelerated regulatory FDA approval for the reduction of proteinuria in adults with primary IgAN at risk of rapid disease progression (generally a urine protein-to-creatinine ratio ≥1.5 g/g) in August 2024.^74^^,^^75^

Most investigational anticomplement inhibitors of the alternative pathway target the FB molecule (Table 3). Selective inhibitors of FB target alternative pathway–mediated cleavage of C3 and specifically block activation of the alternative pathway amplification loop by preventing the formation of the alternative pathway C3 convertase (Figure 1).^244^ In comparison, C3 inhibitors block the complement system at the convergence point of all 3 initiating pathways and the alternative pathway amplification loop.^73^ Similarly, C3aR inhibitors currently in preclinical development, such as SB 290157, are not specific to the alternative pathway and target the common terminal pathway by preventing the inflammatory effects of C3a.^245^

The specific pathophysiology of the disease in question should guide the choice between targeting the alternative or terminal pathways. In alternative pathway-driven CMKDs, alternative pathway inhibition may offer several advantages over broader inhibition of the terminal pathway. For example, in diseases where alternative pathway overactivation is the primary driver, such as C3G or aHUS,^5^^,^^6^ inhibitors of the alternative pathway address the source of complement dysregulation, which may result in more effective disease control. In addition, alternative pathway inhibition may be more beneficial than terminal pathway inhibition in diseases where C3a and C3b are critical drivers of the proinflammatory response. This has already been demonstrated in the rare blood disorder paroxysmal nocturnal hemoglobinuria, a complement-driven condition unrelated to the kidney, in which extravascular hemolysis, mediated by C3b opsonization of red blood cells, is more effectively addressed by alternative pathway inhibition than by terminal pathway inhibition.^246^ Furthermore, in C3G, available data indicate the viability of alternative pathway inhibition,^74^^,^^221^ whereas the benefits of terminal pathway inhibitors are unclear, with patients showing variable clinical responses.^112^^,^^195^^,^^196^ As a potent amplifier of complement activation, targeting the amplification loop earlier in the complement cascade may offer improved suppression of the downstream inflammatory and fibrotic processes that contribute to disease progression compared with blockade of the terminal pathway.^73^ In comparison, inhibition of the terminal pathway may be of benefit in conditions where the release of C5a and formation of the MAC are the primary drivers of glomerular inflammation and tissue damage.^73^ By blocking the proinflammatory effects of C5a and assembly of the MAC, C5 inhibitors may help to prevent the downstream effects of excessive complement activation in diseases where these pathogenic mechanisms predominate.^73^

For CMKDs in which complement dysregulation is primarily driven by the formation or glomerular deposition of antibodies and/or immune complexes, a key consideration that is likely to shape future treatment regimens is the relative merits of a complement-directed approach, which targets the downstream glomerular injury, versus a B-cell–directed or plasma cell–directed approach, which aims to reduce the production of antibodies that drive complement activation.^188^ The benefits of such strategies will require careful evaluation on a disease-by-disease basis. For example, limited case reports suggest a lack of efficacy of B-cell depletion in C3G,^247^ whereas the clinical efficacy of complement-directed alternative pathway inhibition among patients with C3G has been demonstrated.^74^^,^^221^ Conversely, among patients with IgAN, B-cell modulators that reduce B-cell proliferation and survival through the inhibition of a proliferation-inducing ligand and/or B-cell–activating factor have shown early therapeutic promise in phase 1/2 and phase 3 trials.^248^, ^249^, ^250^, ^251^, ^252^, ^253^, ^254^ Importantly, once complement activation and inflammation are established, it is possible that upstream immunomodulation alone may be insufficient to fully control disease activity.^10^ In such cases, a combination approach integrating antibody-directed therapies with complement inhibition may be necessary.^10^ However, further evaluation of the efficacy and optimal application of such treatment strategies within disease-specific contexts is required.

As more complement inhibitors become available, efficacy studies and biomarker-driven approaches could help determine which patients are most likely to benefit from specific types of complement inhibition. Therefore, research comparing alternative and terminal pathway inhibitors, as well as other immunomodulatory therapies, across various CMKDs is essential to refine therapeutic strategies. Furthermore, investigating combination approaches would provide additional insights into optimizing treatment strategies that target multiple aspects of complement dysregulation.

### Summary and Conclusion

CMKDs encompass a diverse range of rare disorders in which the complement system plays a key role in glomerular inflammation and injury.^4^^,^^5^ Patients with CMKDs present across a spectrum of glomerular inflammation and injury that can progress to fibrosis and glomerular scarring associated with declining kidney function. Although the contribution of complement to disease pathogenesis varies by CMKD, the alternative pathway is a key driver of glomerular inflammation and tissue damage through amplification of complement activation, regardless of the initiating pathway.^2^ A growing body of evidence supports a link between the overactivation of the alternative pathway and glomerular inflammation in CMKDs, including the glomerular deposition of key complement regulators and activation fragments, as well as mechanistic insights from preclinical models. The improved understanding of the role of complement activation in glomerular diseases has paved the way for the development of multiple, targeted complement inhibitors, several of which are now approved for clinical use.^68^^,^^74^^,^^77^, ^78^, ^79^, ^80^ Historically, immunosuppressive therapies have been used in the management of glomerular inflammation.^112^ Novel anticomplement therapies, including those targeting the alternative pathway, represent a promising approach to managing glomerular inflammation and injury, ultimately preventing kidney structure loss associated with CMKDs.^10^^,^^188^

## Disclosure

JB reports research grants from Alexion and Novartis; and has received consultancy fees from Alexion, Apellis, Argenx, Arrowhead, Novartis, and Roche. PG reports research grants from the Novo Nordisk Foundation and the Augustinus Foundation; and has received consultancy fees from Alexion, Annexon, Argenx, Dexoligo, Lundbeck, and Sobi. JF has received consultancy fees and/or honoraria from Alexion, AstraZeneca, Bayer, Biogen, Calliditas, CSL Vifor, GlaxoSmithKline, Novartis, Omeros, Otsuka, Roche, STADApharm, Travere, Vera, and Vertex; and serves as the Work Group Co-Chair of the Kidney Disease: Improving Global Outcomes Clinical Practice Guideline for the Management of Glomerular Diseases. RAL is an employee of Stanford University Medical Center; reports travel grants from Biogen and Vera; has received consultancy fees from Alexion, Amgen, Biogen, Calliditas, Deerfield, Novartis, Travere, Vera, and Vertex; and his employer has received research funding from Alexion, BeOne, Biogen, National Institutes of Health, Novartis, Otsuka, Roche, Travere, University of Michigan, University of Pennsylvania, and Vera. HZ is an employer of Peking University First Hospital; and has received consultancy fees from Alpine (Vertex), Alexion, AstraZeneca, Biogen, Calliditas, Chinook (Novartis), George Clinical (Emerald Clinical Trials), Novartis, Otsuka, Roche, Takeda, and Vera.
